# Mature Cystic Teratoma of Douglas’ Pouch: A Rare Entity

**DOI:** 10.7759/cureus.5515

**Published:** 2019-08-29

**Authors:** Pruthwiraj Sethi, Suvendu Purkait

**Affiliations:** 1 Obstetrics and Gynecology, All India Institute of Medical Sciences, Bhubaneswar, IND; 2 Pathology, All India Institute of Medical Sciences, Bhubaneswar, IND

**Keywords:** teratoma, cystic teratoma, teratoma in douglas’ pouch, mature teratoma, benign teratoma

## Abstract

Mature cystic teratoma is one of the more common ovarian neoplasms; however, teratoma in the pouch of Douglas is extremely rare, and the exact etiology is unknown. Here, we report a rare case of mature cystic teratoma of Douglas’ pouch in a 40-year-old woman who had undergone laparotomy. A 15 cm x 15 cm x 10 cm cyst was noted arising from the pouch of Douglas, and the mass was seen separated from both ovaries. Microscopically, the cyst was a mature cystic teratoma not arising from the ovaries.

## Introduction

Mature cystic teratoma is one of the more common variants of ovarian neoplasm, but its presence in the pouch of Douglas is extremely rare. Since the first case of a dermoid cyst in Douglas’ pouch reported by Lefkowitch et al. in 1978 [1], very few cases have been reported to date, and the exact etiology is unknown. Here, we report a case of mature cystic teratoma of Douglas’ pouch in a 40-year-old woman.

## Case presentation

**Figure 1:**
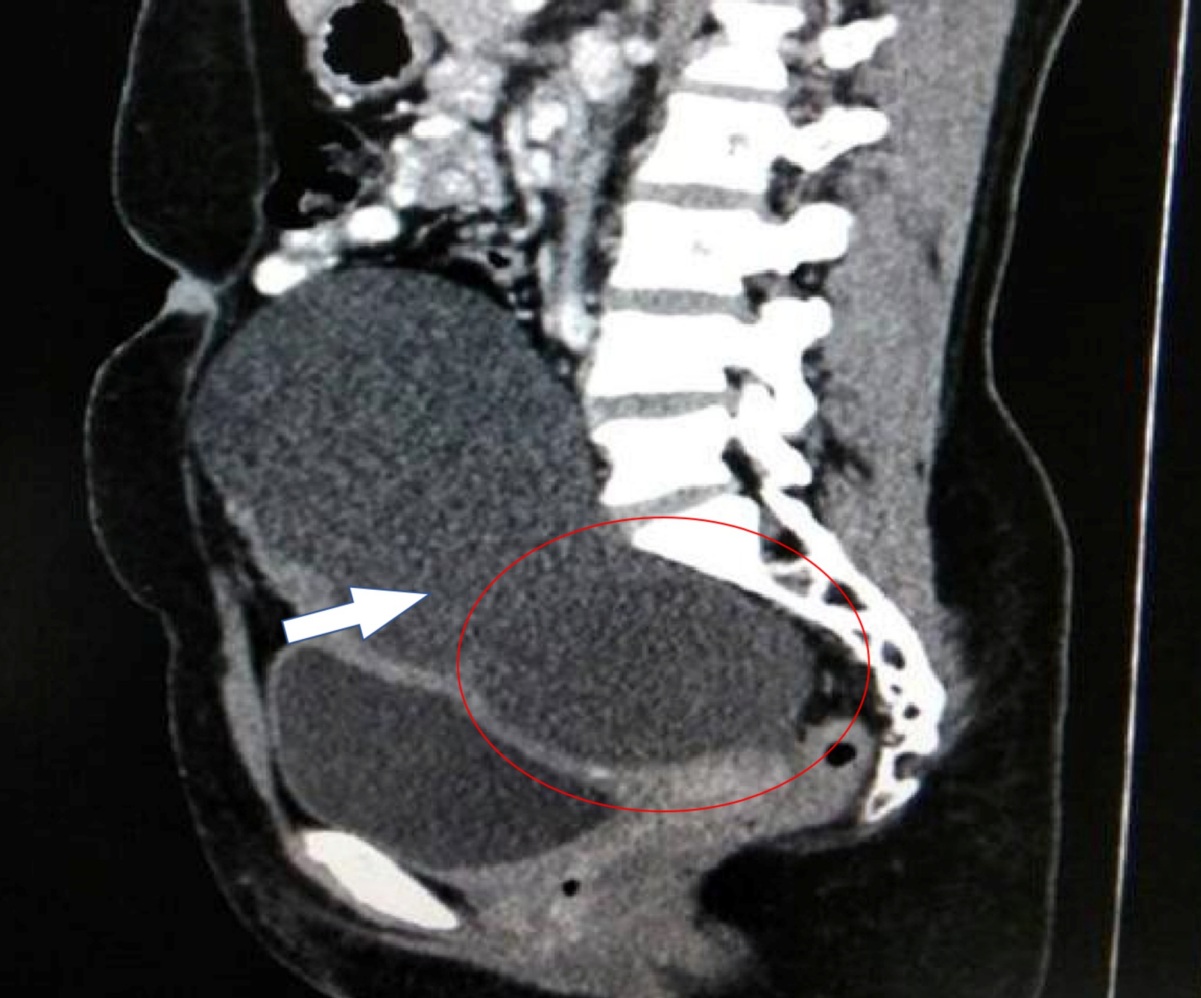
Contrast-enhanced CT scan of pelvis showing cyst arising from Douglas’ pouch.

**Figure 2:**
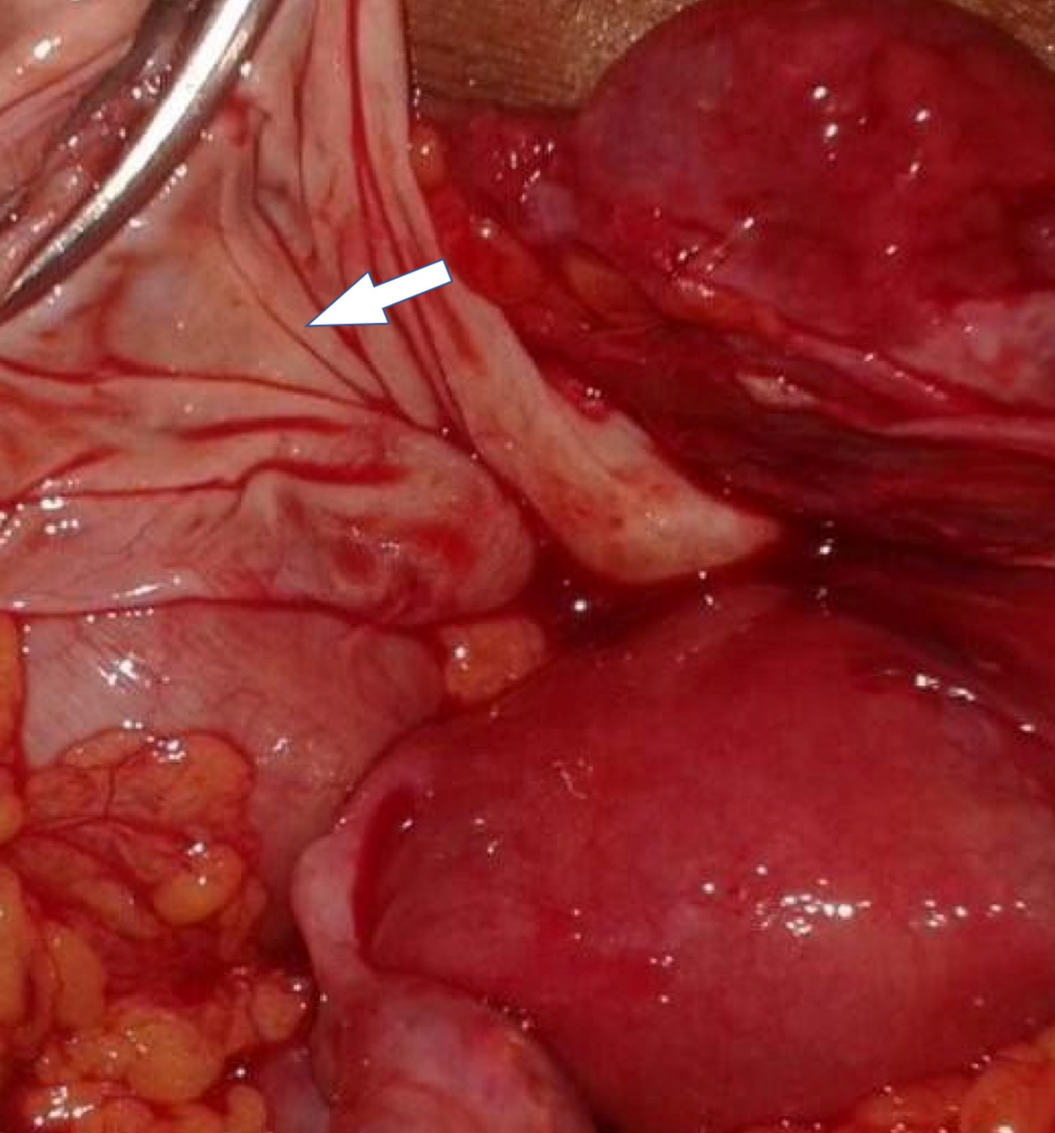
Intraoperative finding showing cyst is free from both ovaries.

**Figure 3:**
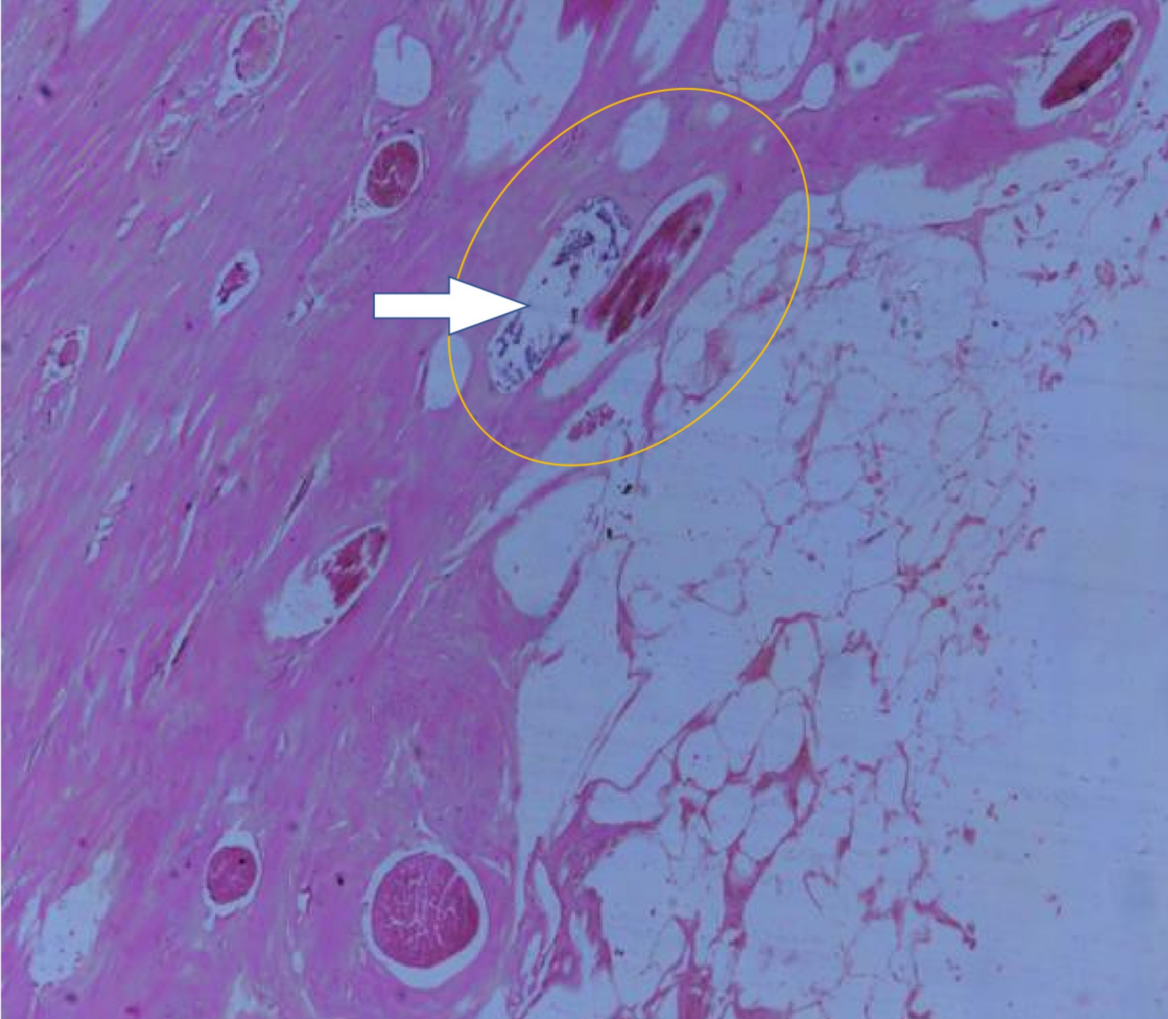
Sections showing fibrocollagenous and adipose tissue with presence of hair follicle and denuded lining (H&E 100x).

A 40-year-old woman presented to the hospital with lower abdominal pain. Her general physical examination was normal. Upon abdominal and pelvic examination, there was a nontender, cystic mass of 16- to 18-week size in the midline. Her uterus was normal in size and free from the mass, and her CA-125 was 11.7 U/mL, and CA 19-9 was 35.26 U/mL. Upon ultrasonography, an ovarian complex mass of 18 cm x 14 cm with thin septation and solid heterogeneous component was seen without increased vascularity. Contrast-enhanced CT scan of the abdomen and pelvis revealed a complex lesion of the left ovary with a defined lesion of 13.1 cm x 10.5 cm x 8.1 cm and a focus of calcification noted in the septa (Figure 1). The diagnosis of ovarian teratoma was made, and a laparotomy was performed. A 15 cm x 15 cm x 10 cm cyst was noted arising from the pouch of Douglas, and both ovaries were normal in size and shape (Figure 2). The cyst wall was ruptured during the operation, resulting in the discharge of approximately 500 mL of thick yellowish-colored fluid, and a bundle of hair was visible inside the cyst. Excision of the cyst wall was performed and a biopsy was taken from both ovaries. The intraoperative and postoperative periods were uneventful. Upon microscopic examination, a mature cystic teratoma not arising from the ovaries was present (Figure 3).

## Discussion

Mature cystic teratomas are most commonly found in the ovaries and account for 20% of all ovarian neoplasms [2]. An extragonadal occurrence is very rare, but when they occur, are primarily in the omentum [3]. The etiology of extragonadal dermoid cysts can be described by three theories. According to the first theory, extragonadal teratomas may arise from displaced germ cells. During early fetal development, germ cells migrate from the yolk sac along the hindgut toward the genital ridge. During this migration, germ cells become arrested between the yolk sac endoderm and the dorsal mesentery and trapped in the retrouterine pouch of Douglas [4]. The second theory states that extragonadal teratomas originate from an ectopic ovary, either due to implantation of ovarian tissue following a surgical procedure or due to inflammation such as pelvic inflammatory disease [5]. The third theory states that extragonadal teratomas are formed due to auto-amputation of an ovarian dermoid and its reimplantation into extra-ovarian sites [6]. In our case, it seems that the teratoma originated from a displaced primordial germ cell, as both ovaries were normal in size and position, and there was no history of operation for an ovarian dermoid. Differential diagnosis of a mature cystic teratoma of Douglas’ pouch includes endometriotic cyst, cystic mesothelioma, and cystic lymphangioma. The preoperative diagnosis of teratoma in Douglas’ pouch is very difficult; however, a MRI or CT scan of the pelvis may sometimes provide a preoperative diagnosis. In our case, it was diagnosed intraoperatively and confirmed by histopathological findings.

## Conclusions

As the incidence of mature cystic teratoma of Douglas’ pouch is extremely rare, the clinical diagnosis can be a challenge. It may provide insights into the differential diagnosis of an ovarian neoplasm.
